# Is only-child status associated with a higher blood pressure in adolescence? An observational study

**DOI:** 10.1007/s00431-022-04800-5

**Published:** 2023-01-20

**Authors:** Pauline Marie Pantke, Christoph Herrmann-Lingen, Aribert Rothenberger, Luise Poustka, Thomas Meyer

**Affiliations:** 1 Klinik für Psychosomatische Medizin und Psychotherapie, Universität Göttingen, Göttingen, Germany; 2 Deutsches Zentrum für Herz-Kreislauf-Forschung (DZHK), Standort Göttingen, Von-Siebold-Str. 5, 37073 Göttingen, Germany; 3 Klinik für Kinder- und Jugendpsychiatrie und Psychotherapie, Universität Göttingen, Göttingen, Germany

**Keywords:** Birth order, Only-child status, Blood pressure, Quality of life, Adolescents

## Abstract

Growing up with siblings may affect quality of life as well as hemodynamic parameters including blood pressure. Using weighted data from the nationwide and representative German KiGGs study, we assessed the relationship between only-child status, birth order, and arterial blood pressure in a cohort of 7311 adolescents aged between 11 and 17 years. Our data showed that only-children had the highest mean arterial blood pressure (87.3 ± 8.4 mmHg) as compared to first-born (86.3 ± 8.0), middle-born (86.4 ± 8.7), and youngest-born siblings (86.6 ± 8.2; *p* = 0.012). The two groups of only-children and first-borns differed significantly with respect to their age- and sex-specific*, z*-scored data for systolic *(p* = 0.047), diastolic (*p* = 0.012), and mean arterial blood pressure (*p* = 0.005). Linear regression models with blood pressure recordings as dependent variable adjusted to age, migration background, and age- and sex-specific *z*-scores of body-mass index confirmed that only-child status was an independent predictor of a higher diastolic blood pressure (*p* = 0.037). A similar result was observed for mean arterial blood pressure (*p* = 0.033), whereas systolic blood pressure was not associated with only-children status (*p* = 0.258).

*Conclusion*: In summary, we found a significant and positive relationship between only-child status and blood pressure, with the highest recordings in only-children and the lowest in first-borns. Models adjusted for relevant clinical confounders demonstrated slightly higher blood pressure in only-children compared to first-borns, who are both in an alpha birth order. Although these blood pressure differences were statistically significant, they have limited, if any, clinical meaning in this age group.

**Table:** 

**What is Known:** *• In children and adults, blood pressure is significantly higher in only-children compared to children with siblings. However, it is unclear whether there are also similar blood pressure differences in adolescents and between only-children and first-borns.*	
**What is New:** *• Among adolescents in the alpha birth order, growing up as an only-child is associated with significantly higher mean blood pressure than among first-borns living with younger siblings.*

**Supplementary Information:**

The online version contains supplementary material available at 10.1007/s00431-022-04800-5.

## Introduction

A few studies have shown that, in both children and adults, blood pressure was positively associated with higher quality of life and lower levels of distress [1–4]. In 1472 adult participants from the Buffalo Blood Pressure Study conducted in the year 1961, it was demonstrated that those who had grown up without siblings had higher systolic as well as diastolic blood pressure and a higher prevalence of hypertension than those with siblings [5]. Another study reported a continuous inverse relation between the number of siblings and mean blood pressure in a considerably younger cohort of 3591 children from the UK aged from 5 to 7.5 years with the highest blood pressure in participants with no siblings [6, 7]. Given that the link between blood pressure and sibling status was observed already during the first decade of a child’s life, the authors ruled out increased alcohol consumption as an underlying mechanism leading to elevated blood pressure. In a small sample of Brazilian adolescents, the combined group of only-children and first-borns with siblings displayed significantly higher blood pressure than later-borns, which was attributed to variability in early growth and reduced physical activity [8].

Only-children and first-borns may experience different psychosocial developmental conditions in their families during a highly sensitive period of life. For example, first-borns with siblings must cope with complex family interactions, may receive fewer parental resources, and have more responsibilities, while only-children may be faced with overprotection control. So far, recent studies on blood pressure and psychosocial risk or distress in children and adolescents found only limited support for any influence of specific psychosocial factors on blood pressure [9, 10].

In a previous paper from the KiGGS study, we demonstrated a significant link between lower levels of blood pressure and the diagnosis of ADHD [11]. The influence of the more general aspect of quality of life, however, was not yet considered. We hypothesize that, similar to infant and adult populations, also in adolescent only-children blood pressure is significantly higher than in adolescents with siblings. Given the known association between blood pressure and health-related quality of life, we additionally assessed whether quality of life is mediating a putative relation between only-child status and mean arterial blood pressure [1–4]. The aim of this paper was to evaluate the relationships between blood pressure, sibling status and birth rank in data from the nationwide German Health Interview and Examination Survey for Children and Adolescents (Kinder- und Jugendgesundheitssurvey, KiGGs).

## Methods

### Design of the KiGGs study

Data for this post-hoc analysis were obtained from the public use file of the KiGGs study, which was conducted by the Robert Koch-Institute, Berlin, between 2003 and 2006. The aim of the KiGGs study was to obtain cross-sectional data on the physical and mental health using a representative sample of German children and adolescents. Participants were randomly selected from the population registration offices of 167 towns and municipalities scattered across Germany that served as study sites. The KiGGs baseline survey had a response rate of 66.6%, resulting in a total cohort of 17,641 participants aged between 0 and 17 years. The present analysis considers exclusively participants between 11 and 17 years of age, as this group provided self-ratings for quality of life as well as emotional symptoms and behavioral problems. The total study cohort was classified into the two groups of only-children and those with siblings, who were further categorized into first-, middle-, and youngest-borns. Seventy-eight KiGGS study participants who had twin siblings were excluded from the analysis. To allow population-based conclusions, a weighting factor was calculated from the entire study cohort (0 to 17 years of age) to correct for deviations from the normal distribution for age, sex, region, nationality, and educational level of parents. The local site teams comprised five members led by a physician experienced in pediatrics. The KiGGs study was commissioned and funded by the Federal Ministry of Health (BMG) and the Federal Ministry of Education and Research (BMBF). It was approved by the Institutional Review Board of the Charité, University Medicine Berlin, and the German Federal Office for Data Protection. Written informed consent for participation in the study was obtained from the parents and, in addition, from children over 14 years of age. The following study protocol was developed in accordance with the World Medical Association Declaration of Helsinki. 

### Epidemiologic and clinical assessment

The study included detailed physical and medical examinations, a psychometric assessment, and numerous blood and urine tests. In a computer-assisted interview, each participant was asked individually about his or her medical and psychiatric history. Height was recorded with a portable Harpenden stadiometer and weight with a calibrated electronic scale (SECA, Birmingham, UK). From these anthropometric data, body-mass index was calculated (body weight in kg divided by height in meters squared). Socioeconomic status (SES) was assessed according to Winkler, based on the educational levels, professional qualifications, and job-related positions of the parents, as well as on the net income of the household [12]. Study participants were considered migrants when both parents were immigrants and of non-German nationality, at least one parent was not born in Germany and had immigrated from a foreign country or if the study participants themselves had immigrated from abroad. Study participants were classified as confirmed attention-deficit/hyperactivity disorder (ADHD) in case of a physician-based diagnosis, or they were grouped as suspected ADHD if they had either a clinical diagnosis or a score of ≥ 7 on the parent-rated hyperactivity-inattention subscale of the Strengths and Difficulties Questionnaire (SDQ) [13]. Self- and parent-rated quality of life was measured using the well-validated Children’s Quality of Life Questionnaire (KINDL-R), achieving a sufficient reliability by Cronbach’s *α* ≥ 0.70. In general, the KINDL-R follows a generic approach and can thus be used to assess quality of life in both healthy and diseased children [14]. The KINDL-R questionnaire consists of 24 Likert-scaled items assigned to the following six dimensions: physical well-being, emotional well-being, self-esteem, and everyday functioning at school, in the family and with friends. For each item, it can be chosen between the five categories never, seldom, sometimes, often, and always, which allow the calculation of a total sum score between 0 and 100, with 100 representing the highest health-related quality of life. To screen for psychopathological symptoms, the well-validated Strengths and Difficulties Questionnaire (SDQ) was employed, which is used to identify emotional and behavioral problems in children and adolescents [15, 16]. The SDQ measures emotional problems, conduct problems, hyperactivity, peer relationship problems, and prosocial behavior on several subscales. Each of the five subscales is covered by five items which can be chosen between 0 = not true, 1 = somehow true, or 2 = certainly true. By summing the scores, except for the one for prosocial behavior, a total problem burden can be calculated.

### Blood pressure recordings

Blood pressure was measured non-invasively using the Datascope Accuratorr Plus sphygmomanometer with a portable monitor (SOMA Technology, Bloomfield, CT, USA), providing accurate blood pressure measurements in the brachial artery by detecting oscillometric pulsations during cuff deflation [17, 18]. The device meets both the British Hypertension Society (BHS) and the American Association for the Advancement of Medical Instrumentation (AAMI) criteria and is listed in the recommendations of the European Society of Hypertension (ESH) [19]. After 5 min of rest, two consecutive recordings of systolic, diastolic, and mean arterial blood pressure were obtained from each participant. Recordings were performed in a sitting position with the forearm in supination, as described [18]. Depending on the circumference of the subject’s right upper arm, inflatable cuffs were available in four different sizes (6 × 12 cm, 9 × 18 cm, 12 × 23 cm, and 17 × 39 cm) to cover two-thirds of the length of the upper arm from the axilla to the antecubital fossa. According to the manufacturer, the mean error of the Datascope Accuratorr Plus device for systolic and diastolic values is less than ± 5 mmHg with standard deviations of no more than ± 8 mmHg. Given that, in an adolescent population, body-mass index and blood pressure are both developmentally regulated, we used their sex- and age-specific, *z*-transformed scores and not the absolute raw values in all multivariate analyses.

### Statistical analysis

Anonymized data from the Robert Koch-Institute public use file were entered into a computer-assisted database. Statistical analysis was performed with the software Statistical Package for the Social Sciences (SPSS, IBM, New York, USA, version 26), running on a personal computer. Descriptive statistics for numerical data were expressed as means and standard deviations, while categorical data were expressed as frequencies and percentages. Chi-square test for categorical variables was used to assess significant differences between the groups. First-, middle-, and youngest-born children or children with siblings were compared to only-children using analysis of variance (ANOVA) and Student’s *t* test, respectively. Pearson’s correlation test was employed to establish the assumed correlations between mean arterial blood pressure and the self-rated KINDL-R score. Blood pressure measurements with two independent recordings were available for the vast majority of study participants (99.9%), and there were no missing values for migration status. Given this completeness of data, we decided not to use an imputation method but to perform a complete cases analysis, as recommended in the literature [20]. In order to test for independent predictors of arterial blood pressure, a set of linear regression models was calculated using age- and sex-specific, *z*-transformed scores of blood pressure recordings as the dependent variable and only-child status as independent variable adjusted to age, migration background, and z-scores of body-mass index as confounders. The coefficients of determination (*R*^2^) are given in these models as a statistical measure for the percentage of the variance in the dependent variable that the independent variables were collectively explaining. When performing the regression models, we routinely checked the assumptions of normality, linearity, homoscedasticity, and absence of multicollinearity. To determine whether the residuals were normally distributed, a normal predicted probability (P-P) plot was created using the statistical program SPSS. The assumptions were validated by inspection of the resulting graphs. Multicollinearity was tested using correlation coefficients. In all tests, statistical significance was defined as *p* = 0.05.

## Results

### Comparison between adolescents with and without siblings

In the total study cohort of KiGGs participants aged between 11 and 17 years, there were 1013 only-children and 6298 siblings (Supplemental Fig. 1). The two groups did not differ with respect to age (*p* = 0.773), sex (*p* = 0.210), body-mass index (*p* = 0.084), and socioeconomic status (*p* = 0.267; Table 1). Participants with an immigrant background were significantly less likely to have no siblings (odds ratio = 0.6; *p* = < 0.001). In the comparison between only-children and siblings, there were no significant differences with respect to confirmed or suspected attention-deficit/hyperactivity disorder (ADHD) diagnosis (*p* = 0.462 and *p* = 0.088). In the self-completed KINDL-R questionnaire, only-children achieved higher total scores compared to children with siblings, indicating a better quality of life (73.4 ± 9.8 vs. 72.5 ± 10.4, *p* = 0.016, Table 1), but scored only insignificantly lower on the self-rated Strengths and Difficulties Questionnaire (*p* = 0.347). Furthermore, there was a significant difference in self-rated quality of life with respect to birth order. First- (73.1 ± 10.4), middle- (71.2 ± 10.5), and youngest-born children (72.6 ± 10.2) had a lower self-rated KINDL-R score than children without siblings (73.4 ± 9.8, *p* < 0.001; Table 1). Demographic and clinical data, including comparison between only-children and children with siblings, are reported in Table 1.

**Table 1:** Characterization of the weighted KiGGs study cohort aged between 11 and 17 years, including the two comparisons between only-children and children with siblings (*p* value 1) and the four groups of only-children, first-, middle-, and youngest-borns (*p* value 2)

	Total study cohort (*n* = 7311)	Only-children (*n* = 1013)	Total siblings (*n* = 6298)	*P* value	First-born children (*n* = 2417)	Middle-borns (*n* = 1141)	Youngest-borns (*n* = 2739)	*P* value
Age (years)	14.6 ± 2.0	14.6 ± 2.0	14.6 ± 2.0	0.773	14.6 ± 2.0	14.7 ± 2.0	14.6 ± 2.0	0.516
Sex (male, %)	51.0	52.8	50.7	0.210	49.5	48.2	52.7	0.017
Migration background (%)	15.5	10.3	16.4	< 0.001	16.7	20.1	14.6	< 0.001
Body-mass index (kg/m^2^)	21.1 ± 4.1	21.3 ± 3.9	21.1 ± 4.1	0.084	21.0 ± 3.9	21.1 ± 4.3	21.1 ± 4.2	0.149
Socioeconomic status	11.5 ± 4.4	11.3 ± 3.9	11.5 ± 4.5	0.267	11.7 ± 4.4	11.0 ± 4.6	11.5 ± 4.4	< 0.001
Confirmed ADHD (%)	5.6	6.1	5.5	0.462	5.2	4.6	6.2	0.142
Suspected ADHD (%)	9.8	11.4	9.6	0.088	8.8	8.9	10.6	0.034
Self-rated KINDL-R	72.7 ± 10.3	73.4 ± 9.8	72.5 ± 10.4	0.016	73.1 ± 10.4	71.2 ± 10.5	72.6 ± 10.2	< 0.001
Self-rated SDQ	10.0 ± 4.5	9.8 ± 4.4	10.0 ± 4.5	0.347	9.7 ± 4.6	10.4 ± 4.5	10.1 ± 4.5	0.001
Parent-rated SDQ HA ≥ 7 (%)	6.4	8.0	6.1	0.032	5.6	6.5	6.5	0.070
Self-rated SDQ HA ≥ 7 (%)	8.2	7.8	8.2	0.711	7.0	8.2	9.3	0.024
25(OH)D (nmol/l)	44.0 ± 27.5	42.8 ± 24.7	44.2 ± 28.0	0.234	43.8 ± 24.6	42.7 ± 25.3	45.1 ± 31.5	0.173
Magnesium (mmol/l)	0.88 ± 0.07	0.88 ± 0.07	0.88 ± 0.07	0.988	0.88 ± 0.07	0.88 ± 0.07	0.88 ± 0.07	0.599

*ADHD* attention-deficit/hyperactivity disorder, *BP* blood pressure, *KINDL-R* children’s quality of life questionnaire, *SDQ HA* Strengths and Difficulties Questionnaire hyperactivity score

### Relationship between sibling status and blood pressure

Mean arterial blood pressure was significantly higher in only-children than in children with siblings (87.3 ± 8.4 vs. 86.5 ± 8.2 mmHg, *p* = 0.002, Table 2). Likewise, also the diastolic blood pressure was higher in only-children (69.0 ± 7.5 vs. 68.2 ± 7.6 mmHg, *p* = 0.003). There was also a trend for higher systolic blood pressure in the only-children group (115.4 ± 11.1 vs. 114.7 ± 10.9 mmHg, *p* = 0.057). Only-children showed the highest recordings for mean arterial blood pressure, followed by youngest-borns (86.6 ± 8.2 mmHg) and middle-borns (86.4 ± 8.7 mmHg, *p* = 0.002), while the lowest mean blood pressure was recorded in first-born children with siblings (86.3 ± 8.0 mmHg, *p* = 0.012, Table 2). A Tukey post-hoc test from this ANOVA result confirmed that the difference between the two groups of only-children and first-borns was significant (*p* = 0.013, 95%-confidence interval [95%-CI] = 0.14–1.83). For diastolic blood pressure, highest values were reached by only-children, followed by youngest-, first- and at last middle-born children (*p* = 0.011, Table 2). The same pattern was observed also for systolic blood pressure, with the highest recordings found in adolescents without siblings and the lowest values found in first-borns, although this did not reach the significance level (*p* = 0.057).

**Table 2:** Comparisons of blood pressure recordings in adolescent only-children and children having grown up with siblings in relation to birth order. The results demonstrate raw data from the KiGGs study cohort aged between 11 and 17 years, including the two comparisons between only-children and children with siblings as well as the four groups of only-children, first-, middle- and youngest-borns

	Total study cohort (*n* = 7311)	Only- children (*n* = 1013)	Total siblings (*n* = 6298)	*P* value	First-born children (*n* = 2417)	Middle- borns (*n* = 1141)	Youngest-borns (*n* = 2739)	*P* value
Systolic BP (mmHg)	114.8 ± 10.9	115.4 ± 11.1	114.7 ± 10.9	0.057	114.3 ± 10.5	114.8 ± 10.6	114.9 ± 10.9	0.057
Diastolic BP (mmHg)	68.3 ± 7.6	69.0 ± 7.5	68.2 ± 7.6	0.003	68.2 ± 7.4	67.9 ± 7.8	68.3 ± 7.6	0.011
Mean BP (mmHg)	86.6 ± 8.3	87.3 ± 8.4	86.5 ± 8.2	0.002	86.3 ± 8.0	86.4 ± 8.7	86.6 ± 8.2	0.012

Both systolic and mean arterial blood pressure were positively and significantly correlated with the parent-rated quality of life (*r* = 0.030, *p* = 0.012 and *r* = 0.029, *p* = 0.014, respectively). Similarly, there was a trend towards a positive relationship between mean arterial blood pressure and self-rated KINDL-R scores (*r* = 0.021, *p* = 0.074).

Using age- and sex-specific, *z*-transformed data for blood pressure recordings instead of the raw data, these findings were confirmed. Only-children and first-borns differed significantly with respect to the measurement of systolic (*p* = 0.047), diastolic (*p* = 0.012), and mean arterial blood pressure (*p* = 0.005). Despite both groups being in an alpha birth order, growing up without siblings was linked to higher pressure as compared to subjects living with younger siblings.

### Association between only-child status and blood pressure in multivariate models

Given the significant relationship between arterial blood pressure and sibling status in univariate analyses for both raw data and their adjusted *z*-scores, we next computed a set of linear regression models with blood pressure recordings as dependent variable and sibling status as independent variable adjusted to clinically relevant confounders (Table 3). Data showed that sibling status was a significant predictor for diastolic (β = 0.035, 95%-CI = 0.005–0.147, *p* = 0.037) and mean arterial blood pressure (*β* = 0.035, 95%-CI = 0.006–0.145, *p* = 0.033). However, in the similar model, higher systolic blood pressure was no longer associated with only-child status (*p* = 0.258).

**Table 3:** Results from three linear regression models with age- and sex-specific, *z*-scored data of diastolic (model 1), mean (model 2), and systolic (model 3) arterial blood pressure recordings as dependent variable and only-child status as independent variable adjusted to age- and sex-specific z-scores of body-mass index and migration background

	Beta	95%-CI	*p* value
Model 1 for diastolic blood pressure (*r*^2^ = 0.025, *p* < 0.001)			
Age	0.031	[− 0.001; 0.032]	0.067
Body-mass index	0.150	[0.118; 0.185]	< 0.001
Migration background	0.016	[− 0.47; 0.136]	0.345
Only-child status	0.035	[0.005; 0.147]	0.037
Model 2 for mean blood pressure (*r*^2^ = 0.067, *p* < 0.001)			
Age	0.028	[− 0.002; 0.030]	0.090
Body-mass index	0.254	[0.223; 0.289]	< 0.001
Migration background	0.033	[0.002; 0.181]	0.046
Only-child status	0.035	[0.006; 0.145]	0.033
Model 3 for systolic blood pressure (*r*^2^ = 0.106, *p* < 0.001)			
Age	0.023	[− 0.001; 0.032]	0.157
Body-mass index	0.324	[0.118; 0.185]	< 0.001
Migration background	0.031	[− 0.47; 0.136]	0.058
Only-child status	0.018	[− 0.029; 0.108]	0.258

## Discussion

In the present post-hoc analysis using a cohort of 11- to 17-year-old adolescents from the KiGGs study, we observed that arterial blood pressure recordings differed significantly with respect to sibling status. The main finding from our analysis is that adolescents living without siblings showed a slightly but significantly higher diastolic and mean arterial blood pressure than those growing up with siblings. Furthermore, we observed that first-borns from families with younger siblings had the lowest mean blood pressure but reported a higher quality of life than middle- and youngest-born children. Using age- and sex-specific, *z*-transformed blood pressure data, regression models adjusted to clinically relevant confounders confirmed that both diastolic and mean arterial blood pressure recordings were significantly associated with only-child status, whereas, in a similarly adjusted model, systolic blood pressure was no longer linked to only-child status.

Our finding demonstrating higher blood pressure in adolescents without siblings compared to those growing up with younger siblings extends previous studies both published in 1991 that reported a similar relationship in other age groups. Whincup et al. detected higher blood pressure in 5- to 7.5-year-old only-children from a sample in the UK, highlighting the early onset of this association [6]. A similar relation was found by Trevisan and colleagues in adults ranged in age between 20 and 70 years [5]. The authors hypothesized that psychological characteristics linked to only-child status, including reduced sociability, increased parental attention, and altered social support, may contribute to this association.

It is a common assumption that growing up without siblings brings several disadvantages in terms of social and affiliative behaviors and interactions with other children. Using a sample of 139 primary school-aged children, it was found that only-children were less popular with their classmates and more prone to aggressive behavior [21]. In a study regarding mental health problems, increased submissiveness towards their parents and maternal overprotective attitudes were more frequently observed in only-children [22]. Also, the presence of siblings was considered advantageous for the general physical fitness of children [23]. However, in our study adolescents without siblings rated their quality of life significantly higher than those with siblings. In a previous paper from the KiGGs study, we reported that blood pressure was positively associated with improved well-being and less distress [3]. This may result from a positive feedback loop between repressed emotions leading to elevated blood pressure which in turn has a stress-buffering function. By experiencing social difficulties in dealing with other children of the same age, only-children may acquire such repression of emotions already at an early age in their development. As parents of only-children probably often have high expectations of their child and make strict demands, only-children may be more achievement-orientated than children with siblings at the expense of chronic stress and higher blood pressure.

Our data showed that among children with siblings there is a gradual increase in mean blood pressure recordings from first-borns to middle- and youngest-born children. In contrast, several studies using smaller sample sizes reported higher blood pressure levels in first-born subjects or those with earlier birth ranks [8, 24–26]. This discrepancy may result from the fact that, in these smaller-sized samples, only-children and first-borns from families with more than one child were combined in one category, while in our large-sized sample we classified along the birth order in the four discrete groups of only-children, first-, middle-, and youngest-born children. Given that first-born and only-children are both the first delivered offspring, any biological theories suggesting changes in the uterine nutrient supply during the first pregnancy as a possible mechanism for linking the birth order to blood pressure can be excluded. In addition, we found that quality of life is a significant mediator in the relationship between sibling status and blood pressure (data not shown).

First-born children as compared to their younger siblings may be at a disadvantage in terms of emotional adjustment due to increased competition for parental attention [27]. They may feel a greater weight of responsibility, for example for their younger siblings, which requires a strong emotional adjustment. Older siblings perceived the parenting styles of their guardians as significantly more rejecting and they may have a greater pressure to act as a role model for their younger siblings [28]. Our data corroborate ongoing research in this field that suggests greater parental investment in only-children [29]. It was shown that a parenting style perceived negatively by children is harmful with respect to lower psychological resilience, emotion control and goal-focusing skills, particularly for first-born children as compared to only-children [30]. First-born children may experience a deprivation of parental resources resulting from the birth of younger siblings and simultaneously more responsibilities [31]. In line, our data showed that first-borns are less likely to develop symptoms of ADHD when compared to their younger siblings.

The results of the present study need to be considered in light of several limitations. Due to the cross-sectional nature and post-hoc design of the analysis, causal interpretations with respect to the observed associations between the sibling status, blood pressure, and quality of life are not justified. Although blood pressure recordings were performed under highly standardized conditions by trained personnel, the assessment of blood pressure in relation to birth order was not the original aim of the study. Accordingly, ambulatory blood pressure monitoring was not conducted. Furthermore, no data were available regarding the exact number of siblings of the participants, which may complicate the interpretation of our findings. Because of the fact that blood pressure levels were only slightly higher in the group of adolescents who grew up without siblings, this observation has limited, if any, clinical meaning in this age group. However, these minor differences may be more noticeable during the transition from adolescence to early and later adulthood, as blood pressure tends to increase with age.

Nevertheless, our study has also several strengths, which are mainly based on the large-sized and well-characterized sample representative for the German youth. This provides our study with sufficient statistical power and a certain degree of generalizability. However, the observed effects were very small and of no immediate clinical relevance. The standardized assessment of blood pressure, quality of life and psychopathological symptoms using well-validated psychometric instruments represents a further strength.

In summary, in a representative and nationwide cohort of adolescents, we found small but significant associations between living with siblings and blood pressure recordings. Growing up without siblings is linked to a significantly higher diastolic and mean blood pressure in only-children as compared to their first-born counterparts, despite both groups being in an alpha birth order. The underlying physiological mechanisms behind this finding remain to be determined.

## Supplementary Information

Below is the link to the electronic supplementary material.

Supplementary file1 (TIF 223 KB)

## Data Availability

Data underlying this article will be shared on reasonable request to the corresponding author.

## Abbreviations

ADHD: Attention-deficit/hyperactivity disorder
ANOVA: Analysis of variance
KiGGs: German Health Interview and Examination Survey for Children and Adolescents
KINDL-R: Children’s Quality of Life Questionnaire
SDQ: Strengths and Difficulties Questionnaire

## References

[1] Winkleby MA, Ragland DR, Syme SL (1988). Self-reported stressors and hypertension: evidence of an inverse association. Am J Epidemiol.

[2] Nyklíček I, Vingerhoets JJ, Van Heck GL (1996). Hypertension and objective and self-reported stressor exposure: A review. J Psychosom Res.

[3] Berendes A, Meyer T, Hulpke-Wette M, Herrmann-Lingen C (2013). Association of elevated blood pressure with low distress and good quality of life: results from the nationwide representative German Health Interview and Examination Survey for Children and Adolescents. Psychosom Med.

[4] Herrmann-Lingen C, Meyer T, Bosbach A, Chavanon ML, Hassoun L, Edelmann F, Wachter R (2018). Cross-sectional and longitudinal associations of systolic blood pressure with quality of life and depressive mood in older adults with cardiovascular risk factors: Results from the observational DIAST-CHF study. Psychosom Med.

[5] Trevisan M, Krogh V, Klimowski L, Bland S, Winkelstein W (1991). Absence of siblings - a risk factor for hypertension?. N Engl J Med.

[6] Whincup PH, Cook DG, Papacosta O, Shaper G, Walker M (1991). Relation of blood pressure to number of siblings. N Engl J Med.

[7] Whincup PH, Cook DG, Shaper AG (1989) Early influences on blood pressure: a study of children aged 5–7 years. BMJ 299:587–591 10.1136/bmj.299.6699.587 PMC1837466 2508814

[8] Wells JC, Hallal PC, Reichert FF, Dumith SC, Menezes AM, Victora CG (2011). Associations of birth order with early growth and adolescent height, body composition, and blood pressure: prospective birth cohort from Brazil. Am J Epidemiol.

[9] Doom JR, Rivera KM, Blanco E, Burrows R, Correa-Burrows P, East PL, Lozoff B, Gahagan S (2020). Sensitive periods for psychosocial risk in childhood and adolescence and cardiometabolic outcomes in young adulthood. Dev Psychopathol.

[10] Olive LS, Abhayaratna WP, Byrne D, Telford RM, Berk M, Telford RD (2020). Depression, stress and vascular function from childhood to adolescence: a longitudinal investigation. Gen Hosp Psychiatry.

[11] Meyer T, Becker A, Sundermann J, Rothenberger A, Herrmann-Lingen C (2017). Attention deficit-hyperactivity disorder is associated with reduced blood pressure and serum vitamin D levels: results from the nationwide German Health Interview and Examination Survey for Children and Adolescents [KiGGS]. Eur Child Adolesc Psychiatry.

[12] Winkler J, Stolzenberg H (1999). Der Sozialschichtindex im Bundes-Gesundheitssurvey [Social class index in the Federal Health Survey]. Gesundheitswesen.

[13] Pinho R, Wang B, Becker A, Rothenberger A, Outeiro TF, Herrmann-Lingen C, Meyer T (2019). Attention-deficit/hyperactivity disorder is associated with reduced levels of serum low-density lipoprotein cholesterol in adolescents. Data from the population-based German KiGGS study. World J Biol Psychiatry.

[14] Ravens-Sieberer U, Bullinger M (1998). Assessing health-related quality of life in chronically ill children with the German KINDL: first psychometric and content analytical results. Qual Life Res.

[15] Rothenberger A, Becker A, Erhart M, Wille N, Ravens-Sieberer U, BELLA Study Group (2008). Psychometric properties of the parent Strengths and Difficulties Questionnaire in the general population of German children and adolescents: results of the BELLA study. Eur Child Adolesc Psychiatry.

[16] Becker A, Wang B, Kunze B, Otto C, Schlack R, Hölling H, Ravens-Sieberer U, Klasen F, Rogge J, Isensee C, Rothenberger A, the BELLA Study Group (2018). Normative data of the self-report version of the German Strengths and Difficulties Questionnaire in an epidemiological setting. Z Kinder Jugendpsychiatr Psychother.

[17] Wong SN, Tz Sung RY, Leung LC (2006). Validation of three oscillometric blood pressure devices against auscultatory mercury sphygmomanometer in children. Blood Press Monit.

[18] Neuhauser H, Thamm M (2007) Blutdruckmessung im Kinder- und Jugendgesundheitssurvey (KiGGS). Methodik und erste Ergebnisse [Blood pressure measurement in the German Health Interview and Examination Survey for Children and Adolescents (KiGGS). Methodology and initial results]. Bundesgesundheitsbl Gesundheitsforsch Gesundheitsschutz 50:728–735 10.1007/s00103-007-0234-6 17514457

[19] O’Brien E, Waeber B, Parati G, Staessen J, Myers MG (2001). Blood pressure measuring devices: recommendations of the European Society of Hypertension. BMJ.

[20] Graham JW (2009). Missing data analysis: making it work in the real world. Annu Rev Psychol.

[21] Kitzmann KM, Cohen R, Lockwood RL (2002). Are only children missing out? Comparison of the peer-related social competence of only children and siblings. J Soc Pers Relat.

[22] Howe MG, Madgett ME (1975). Mental health problems associated with the only child. Can Psychiatr Assoc J.

[23] Rodrigues LP, Lima RF, Silva AF, Clemente FM, Camões M, Nikolaidis PT, Rosemann T, Knechtle B (2020). Physical fitness and somatic characteristics of the only child. Front Pediatr.

[24] Ayyavoo A, Savage T, Derraik JG, Hofman PL, Cutfield WS (2013). First-born children have reduced insulin sensitivity and higher daytime blood pressure compared to later-born children. J Clin Endocrinol Metab.

[25] Black SE, Devereux PJ, Salvanes KG (2016). Healthy(?), wealthy, and wise: Birth order and adult health. Econ Hum Biol.

[26] Theodore RF, Broadbent J, Nagin D, Ambler A, Hogan S, Ramrakha S, Cutfield W, Williams MJ, Harrington H, Moffitt TE, Caspi A, Milne B, Poulton R (2015). Childhood to early-midlife systolic blood pressure trajectories: early-life predictors, effect modifiers, and adult cardiovascular outcomes. Hypertension.

[27] Jahangard L, Haghighi M, Bajoghli H, Holsboer-Trachsler E, Brand S (2013). Among a sample of Iranian students, adult attention deficit hyperactivity disorder is related to childhood ADHD, but not to age, gender, socioeconomic status, or birth order. An exploratory study. Int J Psychiatry Clin Pract.

[28] Someya T, Uehara T, Kadowaki M, Tang SW, Takahashi S (2000). Effects of gender difference and birth order on perceived parenting styles, measured by the EMBU scale, in Japanese two-sibling subjects. Psychiatry Clin Neurosci.

[29] Wikle JS, Ackert E, Jensen AC (2019). Companionship patterns and emotional states during social interactions for adolescents with and without siblings. J Youth and Adolesc.

[30] Morgan T, Yang S, Liu B, Cao Y (2020). A comparison of psychological resilience and related factors in Chinese firstborn and only children. Asian J Psychiatr.

[31] Reimelt C, Wolff N, Hölling H, Mogwitz S, Ehrlich S, Martini J, Roessner V (2021). Siblings and birth order-are they important for the occurrence of ADHD?. J Atten Disord.

