# Supplementary figures and images for: Is only-child status associated with a higher blood pressure in adolescence? An observational study
Source: Eur J Pediatr. 2023 Jan 20;182(3):1377–84. doi: 10.1007/s00431-022-04800-5 (PMC10023605; doi:10.1007/s00431-022-04800-5)

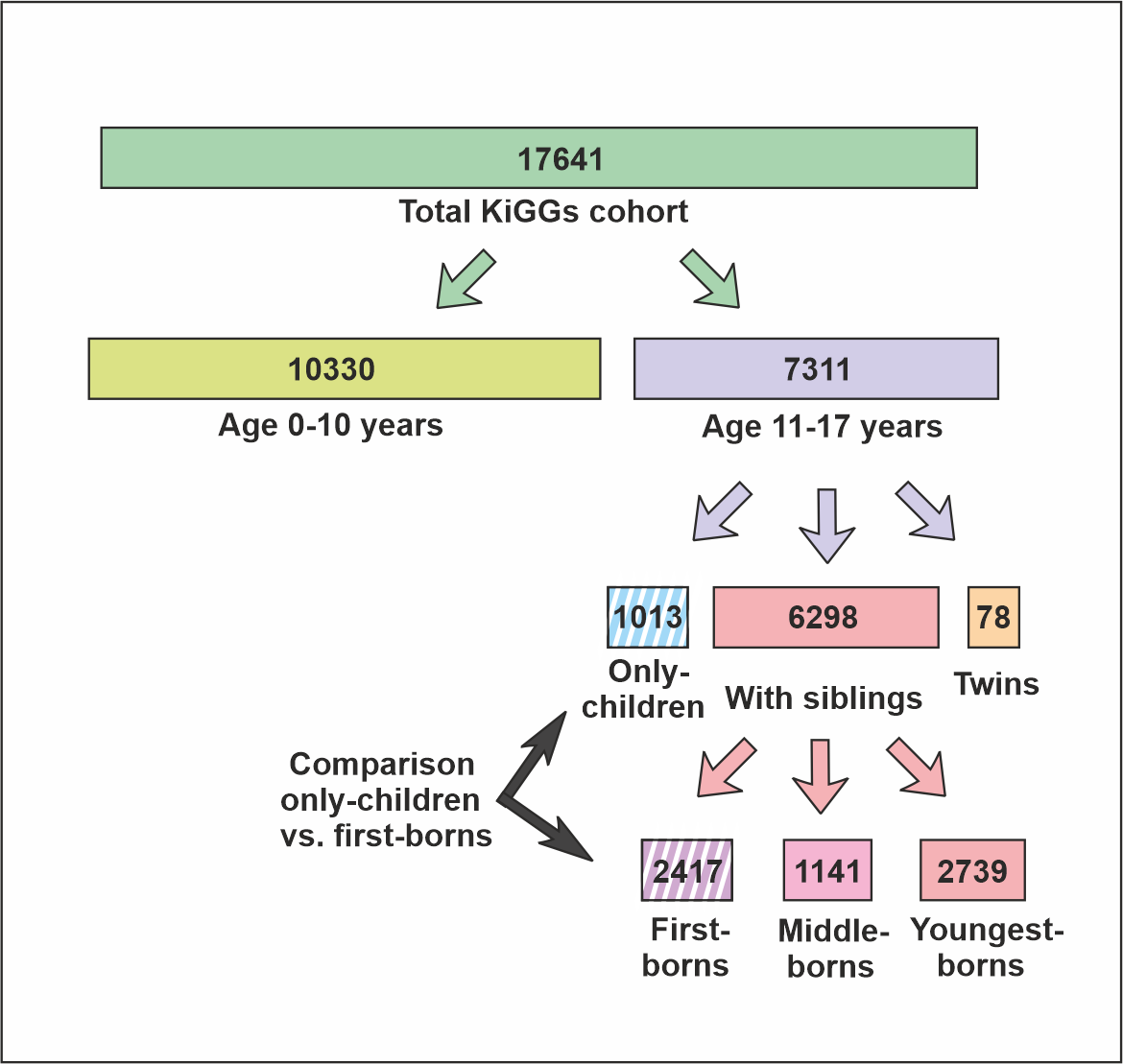

Supplement: Supplementary file 1 — Supplementary file1 (TIF 223 KB) [file 431_2022_4800_MOESM1_ESM.tif]
